# Validation of a genome-wide polygenic score for body mass index in South Asians

**DOI:** 10.3389/fgene.2025.1603542

**Published:** 2025-09-03

**Authors:** Ramesh Menon, Nikhat Khan, Sandeep Charugulla, Akshi Bassi, Pooja Dangare, Akshay Dedaniya, Aakanksha Pant, Rammurthy Anjanappa, Praveena L. Samson, Uthra Satagopan, Sakthivel Murugan, Amol Naikawadi, Vedam L. Ramprasad, Ravi Gupta

**Affiliations:** 1 MedGenome Labs Pvt. Ltd., Bengaluru, Karnataka, India; 2 Genetics Department, Indus Health Plus, Pune, India

**Keywords:** polygenic risk score, obesity, South Asian, genetic screening, validation studies

## Abstract

Obesity is a complex disorder, manifested by the interaction of inherited and environmental factors and modulated by a person’s lifestyle habits. India has witnessed more than a two-fold increase in the number of overweight adults in the last 30 years. The polygenic risk score (PRS) quantitatively measures an individual’s risk for common diseases. The PRS for obesity have been validated in the Caucasian population but not in the South Asian (SAS) population. In this study, we benchmarked and validated the existing genome-wide PRS model of obesity with 2.1 million variants in the SAS population. We analyzed a total of 14,263 individuals from three different South Asian cohorts. We compared the risk score with the body mass index (BMI) categories (underweight, normal weight, overweight, and obese) in all three cohorts. High PRS was associated with increased BMI in all the three cohorts. This study also compared validation results from another population-specific PRS model for the BMI. We conclude that high PRS is associated with high BMI in South Asians. Our study suggests that the PRS score can perhaps be an early predictor of overweight and obesity in the South Asian population.

## Introduction

Obesity is a lifestyle disorder that has seen an alarming rise in recent times. The prevalence of obesity has tripled since 1975, with approximately 650 million adults older than 18 years and nearly 340 million children in the age group 5–18 years having an unhealthy body mass index (BMI) (Jih et al., 2014). It is one of the key factors that increase the risk for diabetes apart from other chronic diseases such as hypertension, heart disease, cancers, and arthritis (Forouzanfar et al., 2016). Notably, a population-based longitudinal study showed that the lifetime diabetes risk was as high as 86% in obese South Asian individuals (Deepa et al., 2015).

Genetic susceptibility to morbid obesity has often been attributed to monogenic mutations in genes such as *FTO, LEPR, MC4R,* and *PCSK1* (Clément et al., 1998; Fawcett and Barroso, 2010; Yeo et al., 1998; Jackson et al., 1997)*.* Unlike monogenic obesity, polygenic obesity is caused by small individual impacts of several genetic variations across the genome. The polygenic risk score (PRS) is by far the most reliable approach to evaluate an individual’s risk for complex diseases and traits. PRS has been successfully demonstrated in cardiovascular diseases, metabolic disorders, neurologic disorders, and various cancer types (Nikpay et al., 2015; Zeinomar and Chung, 2020).

The economic burden that can arise due to obesity and its related health implications is enormous. Hence, identification of the genetic risk for obesity is of profound importance for the prevention and efficient management of the associated conditions. The majority of PRS-based studies on obesity have been conducted in the European population, especially with samples derived from the UK Biobank. However, the impact of obesity polygenic risk markers in the South Asian population (SAS) is still not known. Previously, we reported the validation of the PRS for coronary artery disease (CAD) in SAS cohorts (Wang et al., 2020). In this study, we report the outcome of genome-wide polygenic risk markers for the BMI in South Asian cohorts.

## Results

### South Asian validation cohort and ethnicity assessment

A total of 14,247 samples out of 15,503 were included in the study after removing the samples that failed various QC steps (see methods) (Table 1). Principal component analysis (PCA) was performed for the three cohorts, while keeping the GenomeAsia global populations including the South Asian samples as the reference (Wall et al., 2023; Supplementary Figure S1). We performed PCA separately for the UK Biobank dataset (Affymetrix Axiom) as the genotyping platform is different from other two cohorts (BMI.SAS.1 and BMI.SAS.2), for which the genotype platform is Illumina Infinium GSA version 3. The BMI.SAS.1 (pink color) and BMI.SAS.2 (green color) samples are shown in Supplementary Figure S1A. These samples overlap with the South Asian samples of the GenomeAsia cohort. Similarly, as expected, the BMI.UKB.SAS cohort samples (yellow color) overlaps with SAS samples from GenomeAsia cohort (Supplementary Figure S1B).

**TABLE 1:** Sample summary with sample numbers, the median age with standard deviation, male percentage, and median BMI with standard deviation of the three cohorts included in the study.

Cohort name	Number of samples	Median age (SD)	Male (%)	Median BMI (SD)
BMI.SAS.1	2,992	55 (±10)	84.65	**25.3 (**±**3.9)**
BMI.SAS.2	3,782	39 (±12.3)	62.79	**25.1 (**±**5.5)**
BMI.UKB.SAS	7,473	53 (±8.5)	53.12	**26.6 (**±**4.5)**

In the study cohort, the median BMI increased from 23 to 26 until the age of 38 years (N = 2072, male% = 63.9), stayed more or less the same until the age of 68 years (N = 11,727, male% = 61.6), gradually decreased to 24 in the 68–78-year age group (N = 425, male% = 72), and then finally was 22 in the 78–98-year age group (N = 23, male% = 60.8; Supplementary Figure S2).

### Benchmarking of polygenic risk scores

We benchmarked the performance of two large-sized published BMI–PRS models from Khera et al. (2019) and Yengo and colleagues (Yengo et. al., 2018) in South Asian samples. Comparison of age- and gender-adjusted BMI–PRS obtained from *Yengo et al.* and *Khera et al.* showed Pearson’s correlation of 0.14 and 0.16, respectively, for BMI–PRS and measured BMI (Supplementary Figure S3). The model of *Khera et al.* performed better separately for the three South Asian cohorts (BM1.SAS.1, BMI.SAS.2, and BMI.UKB.SAS) as well. The model of Khera et al. showed a better correlation than the one of Yengo et al., and for the following analysis in this study, we have used the model of Khera et al.

### Validation of the polygenic risk score for BMI in South Asian cohorts

**FIGURE 1:**
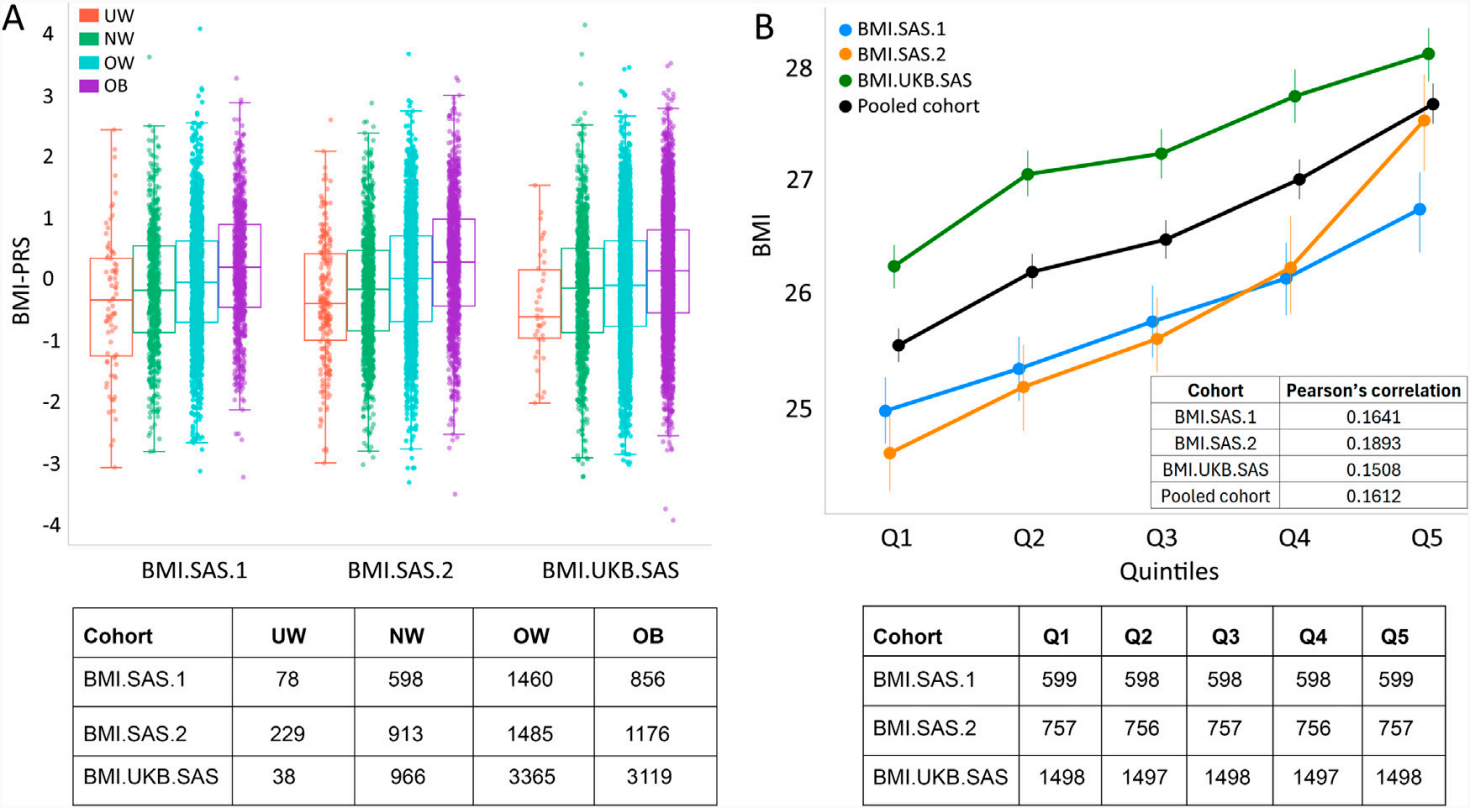
BMI–PRS in various categories and the comparison with measured BMI. **(A)** BMI–PRS distribution in BMI categories in the three cohorts. The number of samples in each BMI category is given in the lower panel (UW = underweight, NW = normal weight, OW = overweight, and OB = obese) for the three cohorts **(B)** Measured BMI *versus* BMI–PRS in quintiles for the three cohorts and the pooled cohort (black color) with Pearson’s correlation of BMI and BMI–PRS for each cohort. The lower panel contains the number of samples in each PRS quintile bins for the three cohorts and the pooled cohort.

The generated age- and gender-adjusted BMI polygenic risk score was compared with different BMI categories (UW = underweight, NW = normal weight, OW = overweight, and OB = obese; Figure 1A). An increasing trend of median BMI–PRS was observed from the underweight category to the obese category across all the three cohorts (Figure 1A). We further divided the individuals of the three cohorts into quintiles (five equal bins) based on the normalized PRS. We observed a steady increase in median BMI for individuals from Q1 to Q5, and the finding is consistent across all three cohorts (Figure 1B). The measured BMI showed a modest correlation with BMI–PRS across all groups, including the pooled cohort. Q-statistics was performed for the pooled cohort, and the heterogeneity was found to be insignificant (p.val < 0.1).

**FIGURE 2:**
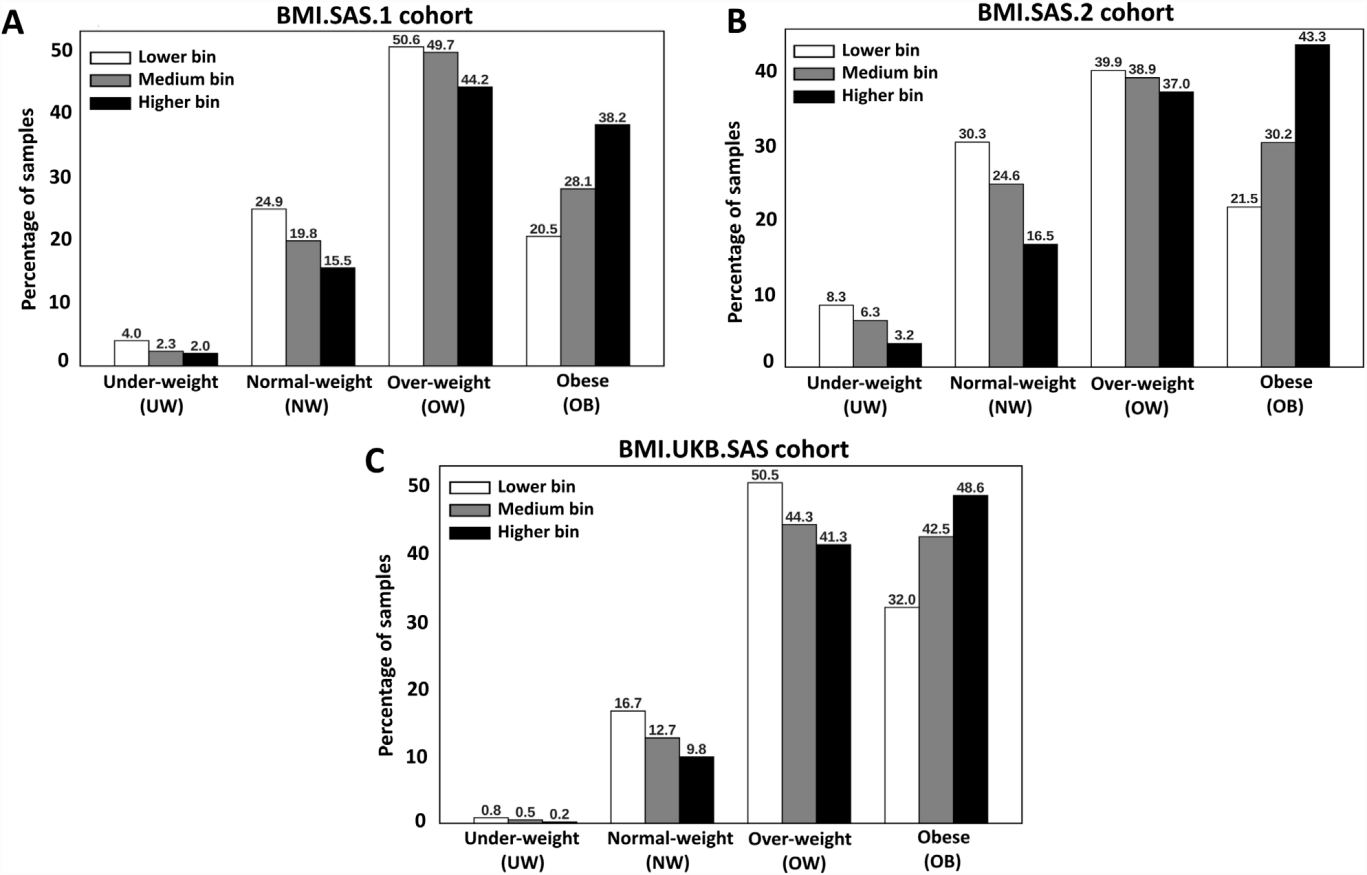
Samples were stratified into three categories, namely, the bottom quintile (Q1, white color), medium quintiles (Q2–Q4, gray color), and the top quintile (Q5, black color), based on the increasing order of the polygenic risk score plotted in the BMI categories for all the three cohorts, where the panels were as follows: **(A)** = BMI.SAS.1; **(B)** = BMI.SAS.2; **(C)** = BMI.UKB.SAS.

We observe a slightly higher BMI for the samples from the BMI.UKB.SAS cohort (Table 1). The increasing trend of the BMI–PRS from Q1 to Q5 was consistent across all three cohorts. Furthermore, we stratified the samples into three groups based on the predicted BMI–PRS score, namely, the lower-risk bin (Q1), medium-risk bins (Q2, Q3, and Q4), and the higher-risk bin (Q5). We then looked at the distribution of these three groups across different measured Asian BMI categories as per the WHO guidelines, namely, underweight, normal weight, overweight, and obese. We observed that more than 80% of the high-BMI risk bin samples are either obese or overweight samples. This was consistently observed in all three cohorts (Figure 2).

**FIGURE 3:**
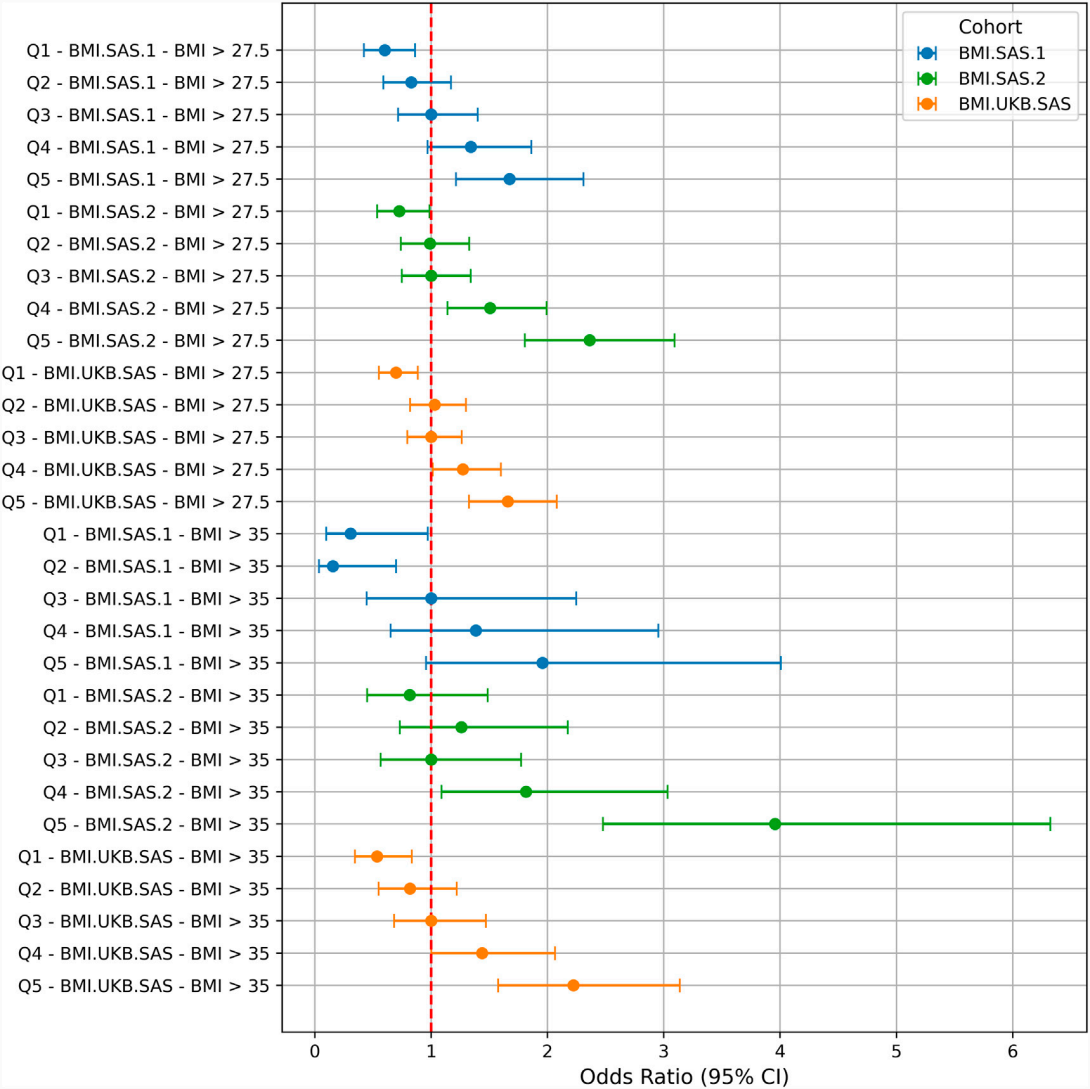
Samples were stratified into quintiles of BMI–PRS scores in the three cohorts (blue color for BMI.SAS.1, green color for BMI.SAS.2, and orange color for BMI.UKB.SAS) for two BMI categories, BMI >27.5 and BMI >35. The odds ratios are given in the x-axis. The dotted vertical line indicates the odds ratio of 1 for middle quintile (Q3), which was taken as the reference for calculating the odds ratios.

We then derived the odds ratios for the obese BMI category (BMI >27.5) and the highly obese category (BMI >35) with respect to the middle quintile (Q3). The samples (BMI >27.5) in Q5 were (Figure 3) associated with a 1.67-, 2.35-, and 1.65-fold increased risk for the BMI.SAS.1, BMI.SAS.2, and BMI.UKB.SAS cohorts, respectively. For the BMI >35 category, the odds ratios were found to be 1.95, 3.95, and 2.22, respectively, in the BMI.SAS.1, BMI.SAS.2, and BMI.UKB.SAS cohorts.

## Discussion

Our study outcome is consistent with the association of high PRS with elevated BMI from other studies on BMI–PRS, suggesting that the combined effects of several genetic variations drive the tendency to gain weight (Khera et al., 2019; Dashti et al., 2022; Hüls et al., 2021). Since there is a huge gap in multi-ethnicity validations, especially in the Asian sub-populations (Choe et al., 2022), in this study, we report an association between increased BMI and high BMI–PRS in 14,247 samples belonging to three distinct South Asian cohorts. By optimizing a BMI–PRS model for South Asians, we observe odds ratios ranging from 1.9- to 3.9-fold increases in PRS for samples with very high BMI (>35). Furthermore, a modest correlation was observed between PRS and the measured BMI. The trend of associations was concordant across the three independent South Asian cohorts. This aligns with the reports of previous studies, which showed the feasibility of the multi-ethnic application of BMI polygenic scores with ancestry corrections and imputation of genotypes with ethnicity-matched large reference panels (Wang et al., 2020; Wall et al., 2023). The odds ratios obtained for samples with BMI >35 in the BMI.SAS.2 cohort (OR = 3.9) were comparable with that reported in the European population (OR = 4.22), which was observed for BMI >40 (Khera et al., 2019).

We observed a two-fold increase in the median BMI through Q1 to Q5 of BMI–PRS categories, which was consistent in all three cohorts. Overweight and obese individuals comprised approximately 80%–90% of the Q5 bin in each of the three study cohorts. Overall, the BMI–PRS was significantly higher in overweight and obese individuals than in normal-weight individuals in all three SAS cohorts, which aligns with the previous findings in the European cohort (Khera et al., 2019). An additional observation was the slightly elevated median BMI in the SAS samples from the UK Biobank than in the other two cohorts.

The rising prevalence of obesity in the South Asian population and the resultant rise in comorbidities, especially cardiovascular diseases, make it imperative to understand the risk factors that result in obesity (Siegel et al., 2014). Coronary artery disease is one of the well-studied examples of the utility of PRSs, where the clinical guidelines recommend ancestry-specific validation before using PRS as a screening tool (Js et al., 2023; O’Sullivan et al., 2022; Abu-El-Haija et al., 2023). A previous study has successfully validated PRS in CAD and has reported that the odds ratios are slightly lower than that of the European population (Wang et al., 2020).

Overall, our study validates and reports the association of high PRS with high BMI, showing the applicability of high PRS as a screening tool for obesity or high BMI in the South Asian population, which can facilitate preventive measures and risk management.

## Materials and methods

### Sample collection

For our BMI study, we obtained data from 15,503 individuals through three different South Asian cohorts (Table 1). The first cohort (BMI.SAS.1) consists of individuals from our previous published study (Wang et al., 2020). The second cohort (BMI.SAS.2) consists of individuals from Indus Health Plus Pvt. Ltd., a preventive healthcare provider located in Pune, India. The consenting individuals went through a health risk assessment telephonic questionnaire conducted by qualified clinicians at Indus Health Plus Pvt. Ltd., where the participants’ present height and weight were asked. Samples with ambiguous or unavailable data were excluded. Furthermore, for a subset of 500 samples, height and weight were measured and compared with the self-reported data. A very high concordance rate was observed between the measured and self-reported data.

The third cohort (BMI.UKB.SAS) was obtained from UK Biobank queried through the ukbREST server (Milton and Hae Kyung, 2019). Individuals with South Asian ancestry from UK Biobank were selected for this study. Additionally, individuals that were distant from the SAS cluster in the ancestry analysis were removed from the respective cohorts (Supplementary Figure S1A, B). Furthermore, only the individuals aged above 18 years were included in this study.

For the BMI.SAS.1 and BMI.SAS.2 cohorts, blood samples (3–5 mL) were collected in EDTA tubes from individuals with informed consent, as per the accepted clinical guidelines and in accordance with the applicable laws from the respective center, and registered with unique identification numbers at MedGenome Labs Ltd., Bangalore. The study using the BMI.SAS.1 cohort was approved by the institutional review boards at each of the recruitment sites. Informed consent with signed forms was collected through the ethics committee for the BMI.SAS.2 cohort.

The BMI.UKB.SAS genotype data were obtained from UK Biobank (UK Biobank ethnicity codes 3001, 3002, and 3003), where BMI and age data were available.

### DNA extraction and genotyping and data quality control

Extraction of genomic DNA from the samples was performed by magnetic separation using the QIASymphony SP system (QIAGEN, Valencia, CA) following the manufacturer’s protocol. The Qubit^®^ dsDNA BR (broad-range) Assay Kit (Thermo Fisher Scientific) was used to quantify the DNA. QIAxpert (QIAGEN, Valencia, CA) and agarose gel were used to assess the quality of DNA. Genotyping was performed using Infinium™ Global Screening Array-24 v3 BeadChip (Illumina, California, United States), which consists of 654,027 genome-wide markers, according to the manufacturer’s protocol (Illumina, California, United States). In this method, 200 ng of genomic DNA was isothermally amplified at 37 °C for 20 h–24 h, enzymatically fragmented, precipitated, resuspended, and loaded onto the BeadChip, which was incubated at 48 °C for 16 h–24 h. Subsequently, the BeadChip was washed and prepared for single-base extension. The BeadChip was scanned on the Illumina iScan System array scanner, as per the protocol given by the manufacturer.

The exported GSA data with phenotypic information such as height, weight, and gender were used to perform quality check through a custom bioinformatics pipeline (MG-ArrayQC tool) using VCFtools version 0.1.14, R package (R version 3.3.2), gdsfmt 1.1.0, and SNPRelate 1.16.0 to generate the call rate, heterozygosity rate, and principal component analysis plots, among others.

### Quality control

Quality assessment was performed on GSAv3 genotyped samples of BMI.SAS.1, BMI.SAS.2, and BMI.UKB.SAS cohorts. Samples with <5% sample and site level missing rate and <95% genotyping rate were removed from the study. Markers with Hardy–Weinberg equilibrium p-value >1e-10 and MAF <0.001 were removed. This was followed by LD pruning for estimating the genetic relationship and PCA. Kinship cutoff of 0.088 was used. PCA was carried out for the samples, and the genotyped samples were projected to the PCA space.

### PRS generation

QC-passed individuals and markers were subjected to genotype imputation using Beagle v5.0 with the GenomeAsia phase 2 (GAv2) imputation reference panel consisting of 6,461 samples, predominantly SAS samples, using the Beagle tool (version 5; Browning et al., 2018; Wall et al., 2023). The 24,687,484 imputed sites were compared with the BMI GWAS summary markers reported in the previous study (Khera et al., 2019).

We obtained the markers with weight from the PGS catalog model (PGSID: PGS000027), which was pre-calibrated (LDpred, ρ = 0.03), comprising 2,100,302 genome-wide markers from the published study in European ancestry. The variants were directly genotyped and imputed using the GenomeAsia reference panel. Of the total 2,100,302 markers in the model, the imputed data were able to cover 83% (1,946,327) of the markers. PRS was generated using the MedGenome pipeline, which uses PLINK v2.0 (Chang et al., 2015) for scoring. Since the summary statistics were derived from the European population, the obtained raw PRS was normalized for the SAS ancestry with GAv2 WGS-500 SAS samples using PCA (PC1–PC5) with FlashPCA R package version 2.6. The PCA cut-offs are provided in Supplementary Table S3.

### BMI–PRS correlation analysis

Pearson’s correlation analysis was performed between the normalized PRS score and BMI categories. The normalized PRS scores and clinical metadata were used for the analysis. Each sample was categorized as underweight (UW), BMI <18.5; normal weight (NW), BMI between 18.5 and 23; overweight (OW), BMI between 23 and 27.5; and obese (OB), BMI >27.5 according to the WHO recommended guidelines for the Asian population (Jih et al., 2014). All the samples in the cohort were sorted in the descending order of the normalized PRS value and then divided into five equal bins. Bin 1 was considered a lower bin, and bin 5 was considered a higher bin. Bins 2, 3, and 4 were considered medium bins.

### Statistical analysis

The age- and gender- adjusted BMI–PRS were generated using the generalized linear model function in *stats* R package version 3.6.2. Nagelkerke R2 estimate of variance explained by the BMI–PRS after covariate adjustment for each cohort and the pooled cohort were calculated using *fmsb* R package version 0.7.6. The Q-statistics was estimated using “gamlss” R package version 5.4.

### PRS validation framework

We followed our earlier framework for the validation of the PRS (Supplementary Figure S4). The upper panel describes the processes involved in the validated PRS in BMI that are deposited in the PGS catalog database (Lambert et al., 2021). The lower panel is the process for the South Asian population-specific validation of the raw PRS generated from the effects’ weights derived model, which was predominantly from BMI studies conducted in populations of European ancestry. The recruited samples were processed, and the missing genotypes were imputed using the GenomeAsia phase 2 reference data comprising 6,461 predominantly South Asian samples (Wall et al., 2023). Then, PCA was performed with the South Asian reference samples. The PC residuals were used to adjust for the ancestry of the South Asian PRS.

## Data Availability

The original contributions presented in the study are publicly available. This data can be found here: https://ega-archive.org/ under accession number: EGAS00001008309.
