# Correction: Validation of a genome-wide polygenic score for body mass index in South Asians

**DOI:** 10.3389/fgene.2025.1708353

**Published:** 2025-11-27

**Authors:** Ramesh Menon, Nikhat Khan, Sandeep Charugulla, Akshi Bassi, Pooja Dangare, Akshay Dedaniya, Aakanksha Pant, Rammurthy Anjanappa, Praveena L. Samson, Uthra Satagopan, Sakthivel Murugan, Amol Naikawadi, Vedam L. Ramprasad, Ravi Gupta

**Affiliations:** 1 MedGenome Labs Pvt. Ltd., Bengaluru, Karnataka, India; 2 Genetics Department, Indus Health Plus, Pune, India

**Keywords:** polygenic risk score, obesity, South Asian, genetic screening, validation studies

In the published article there was an error in the Data Availability statement.

The **Data Availability Statement** was erroneously given as “The unpublished data used in this study has been submitted in the EBI-ENA portal with the accession PRJEB96171”.

The correct **Data Availability Statement** is “The original contributions presented in the study are publicly available. This data can be found here: https://ega-archive.org/ under accession number: EGAS00001008309”.

The original article has been updated.

