# Supplementary material for: Validation of a genome-wide polygenic score for body mass index in South Asians
Source: Front Genet. 2025 Sep 3;16:1603542. doi: 10.3389/fgene.2025.1603542 (PMC12441606; doi:10.3389/fgene.2025.1603542)
Supplement: Supplementary file 1 [file DataSheet1.docx]

**SUPPLEMENTARY MATERIALS**

**1.SUPPLEMENTARY FIGURES**

**
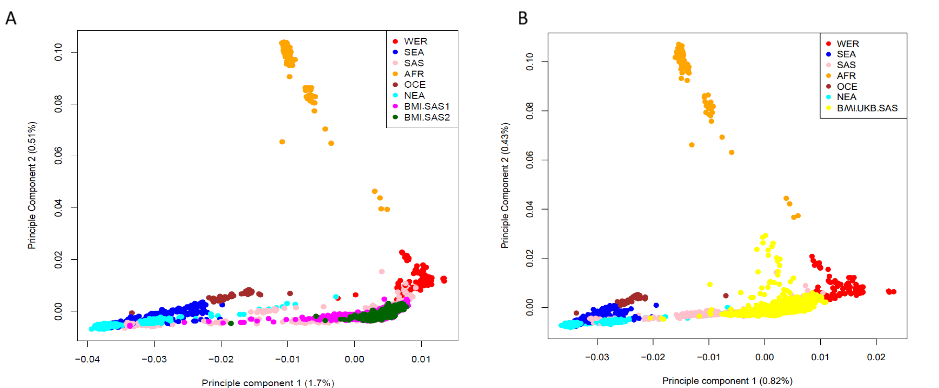
**

**Supp. Fig. S1.** Principal component analysis of the South Asian cohorts. WER=West Eurasian, SEA=SouthEast Asian, SAS=South Asian, AFR=African, OCE=Oceanic, NEA=NorthEast Asian. (A) BMI.SAS.1 and BMI.SAS.2 cohorts, (B) BMI.UKB cohort

**
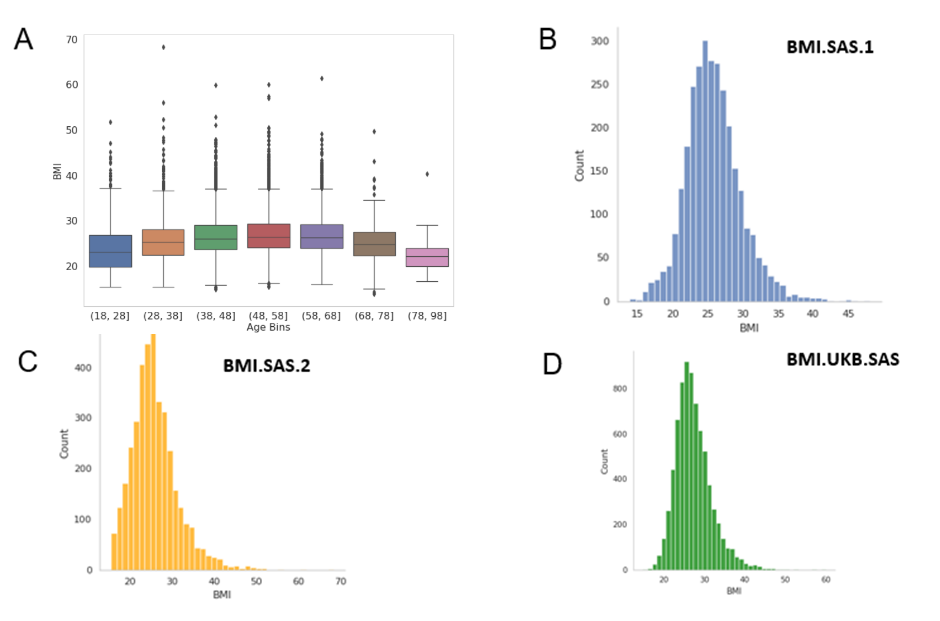
**

**Supp. Fig. S2.** BMI distribution. (A) Age-wise distribution of BMI for combining samples all the three cohorts, (B) BMI.SAS.1, (C) BMI.SAS.2, (D) BMI.UKB.SAS

**
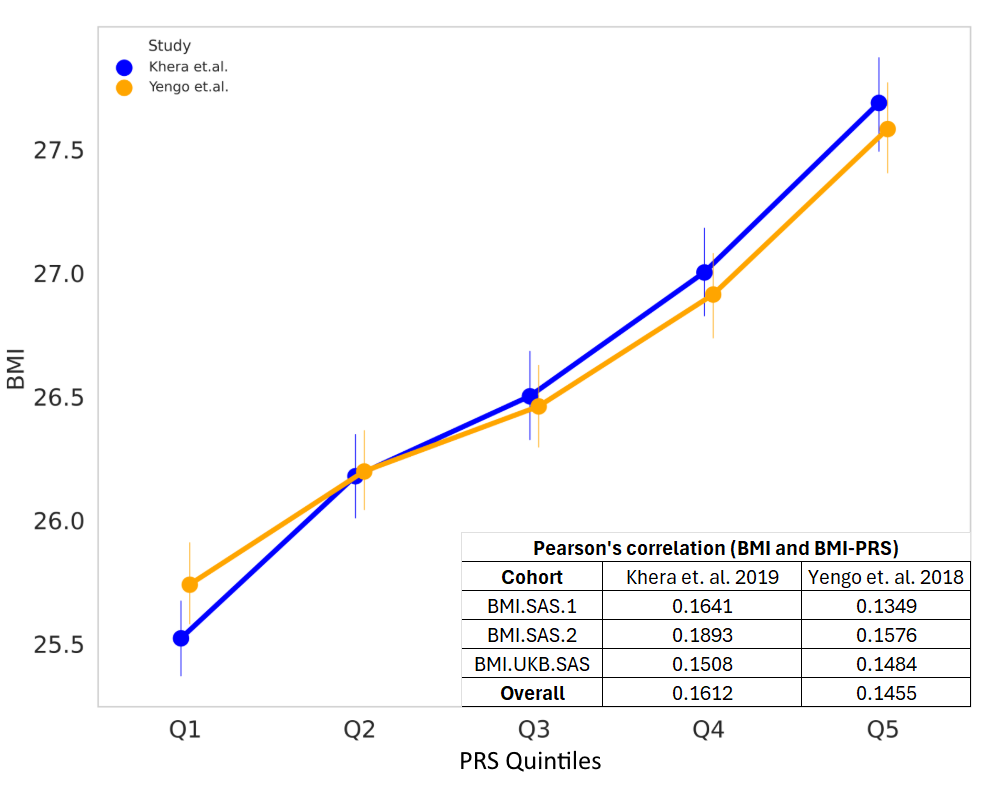
**

**Supp. Fig. S3.** Comparison between the measured BMI and BMI-PRS in and Khera et.al. 2019 (blue color) and Yengo et.al. 2018 (orange color). The Pearson’s correlation values are provided in the inset table in the three cohorts and the pooled cohort.


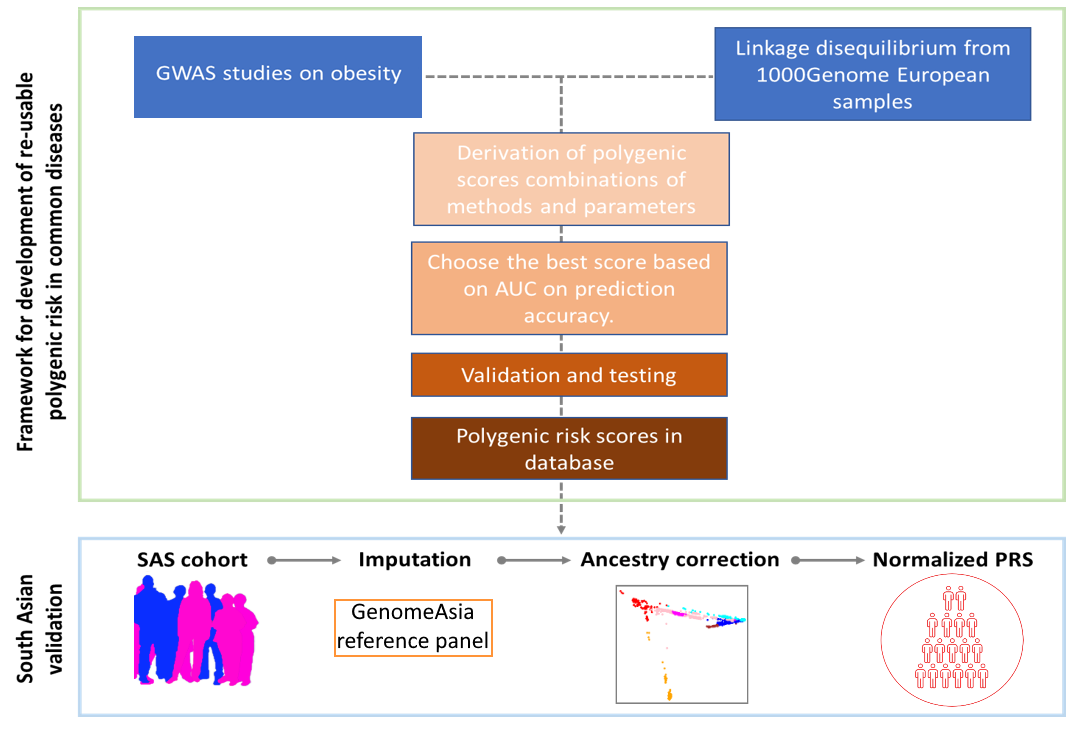


**Supp. Fig. S3.** Framework for validation of polygenic risk score for South Asian population ( Adapted from  doi: 10.1016/j.jacc.2020.06.024)

**2.SUPPLEMENTARY TABLES**

**Supp. Table1.** Nagelkerke’s R2 estimate of variance explained by the BMI-PRS after covariate adjustment for each cohort and pooled cohort.

|  | **No. of samples** | **Nagelkerke’s R2** |
| --- | --- | --- |
| BMI.SAS1 | 2992 | 0.03902395 |
| BMI.SAS2 | 3782 | 0.05659462 |
| UKB.SAS | 7473 | 0.03549629 |
| Pooled cohort | 14247 | 0.04080198 |

**Supp. Table2.** Comparison of correlation between BMI-PRS and measured BMI in various ethnic groups reported in published studies.

| **Study** | **Major ancestry group** | **Correlation coefficient** |
| --- | --- | --- |
| Menon et.al. (Present study) | South Asian | 0.161 |
| Khera et.al. | European | 0.292 |
| Yoon et.al. | Korean | 0.159 |
| Chikowore et.al. | African | 0.131 |

**Supp. Table3.** PC1 and PC2 value used into our study to identify SAS samples.

|  | **BMI.SAS1 & BMI.SAS2** | | **UKB.SAS** | |
| --- | --- | --- | --- | --- |
|  | PC1 | PC2 | PC1 | PC2 |
| **Lower Limit** | -0.03442 | -0.00635 | -0.03134 | -0.00448 |
| **Upper Limit** | 0.00918 | 0.00919 | 0.01043 | 0.00591 |
